# Supplementary material for: Information Thermodynamics and Reducibility of Large Gene Networks
Source: Entropy (Basel). 2021 Jan 1;23(1):63. doi: 10.3390/e23010063 (PMC7824329; doi:10.3390/e23010063)
Supplement: Supplementary file 1 [file entropy-23-00063-s001.pdf]

*entropy* MDPI

# Supplementary Information: Information Thermodynamics and Reducibility of Large Gene Networks

**Swarnavo Sarkar[#], Joseph B. Hubbard, Michael Halter, and Anne L. Plant[*]**

National Institute of Standards and Technology, Gaithersburg, MD 20899, USA
[#]swarnavo.sarkar@nist.gov
[*]anne.plant@nist.gov

## S1. Generating model GRNs

All the model GRNs presented in the main text were generated using the NetworkX package (version 2.5) for Python. Specifically, the barabasi_albert_graph function was used to create graphs using the Barabási-Albert preferential attachment model [1]. The barabasi_albert_graph(n, m) function requires two inputs: (1) the number of nodes in the graph, $n$, and (2) the number of edges connecting a new node to existing nodes in the graph, $m$, and produces an undirected graph. The documentation for the barabasi_albert_graph function[a] fully describes the algorithm for generating the graphs from the inputs $n$ and $m$.

We then convert the undirected Barabási-Albert graph into a directed one. In the NetworkX graph object, an undirected edge between nodes $v_i$ and $v_j$ is equivalent to a directed edge from $v_i$ to $v_j$ and also a directed edge from $v_j$ to $v_i$. We transformed the undirected Barabási-Albert graphs into directed graphs by deleting any edge from $v_i$ to $v_j$ when the node numbers constituting the edge satisfy the condition, $i > j$. This deletion results in every node in the graph having the same in-degree, $m$, without any constraint on the out-degree. The final graph object, $G = (V, E)$, contains vertices numbered from 0 to $n - 1$, and each directed edge $v_i \rightarrow v_j$ in the set $E$ is such that $i < j$. A source node $v_i$ can only send information to node numbers higher than itself. Hence, the non-trivial values in the loss field, $L(i \rightarrow j)$, (as shown in figures 2b and 2c) are confined within the upper diagonal, $i < j$.

## S2. Creating model GRNs with mixtures of up and down regulation

To create the model GRNs with mixture of up and downregulation edges, as shown in Fig. 1b in the main text, we started with the directed Barabási-Albert graphs, $G = (V, E)$, generated as described in Section S1. Let the total number of edges or the cardinality of the set $E$ be $|E|$, and the ratio of downregulation edges to upregulation edges be $\beta$, as defined in the main text. We determined the number of downregulation edges in the graph, $e_{\text{down}}$, as the largest whole number less than or equal to $\beta/(1+\beta)|E|$. Then, we randomly selected $e_{\text{down}}$ edges from the set $E$ and designated them as downregulation edges while specifying the rest as upregulation edges.

The number of nodes that can receive mixed signals, $n_{\text{mixed}}$, was determined by counting the number of nodes in the graph, $G = (V, E)$, that have both an upregulation edge and a downregulation edge directly connecting into it.

---

[a]https://networkx.org/documentation/stable/reference/generated/networkx.generators.random_graphs.barabasi_albert_graph.html#networkx.generators.random_graphs.barabasi_albert_graph

## S3. Accessibility score distributions

Accessibility score for a receiver node $v_j$ is the number of other nodes in the graph that has a path, or a connected set of edges, to send information to it. Increasing the in-degree $m$ creates more paths, and subsequently a receiver node can be accessed by more nodes in the graph. We demonstrate the effect of increasing m by evaluating the distribution of accessibility scores for $m$ =1, 2, and 3 type GRNs with 100 nodes.

For each $m$ value we generated 1000 graphs using the method described in Section S1. Then for each of the graphs we calculated the accessibility of each of the 100 nodes. Therefore, we obtained a total of 1000 × 100 samples of the accessibility score for each value of $m$, which were used to obtain the accessibility score distributions shown in Figure S1. The out-degree distributions for each value of $m$ are shown in Figure S1.

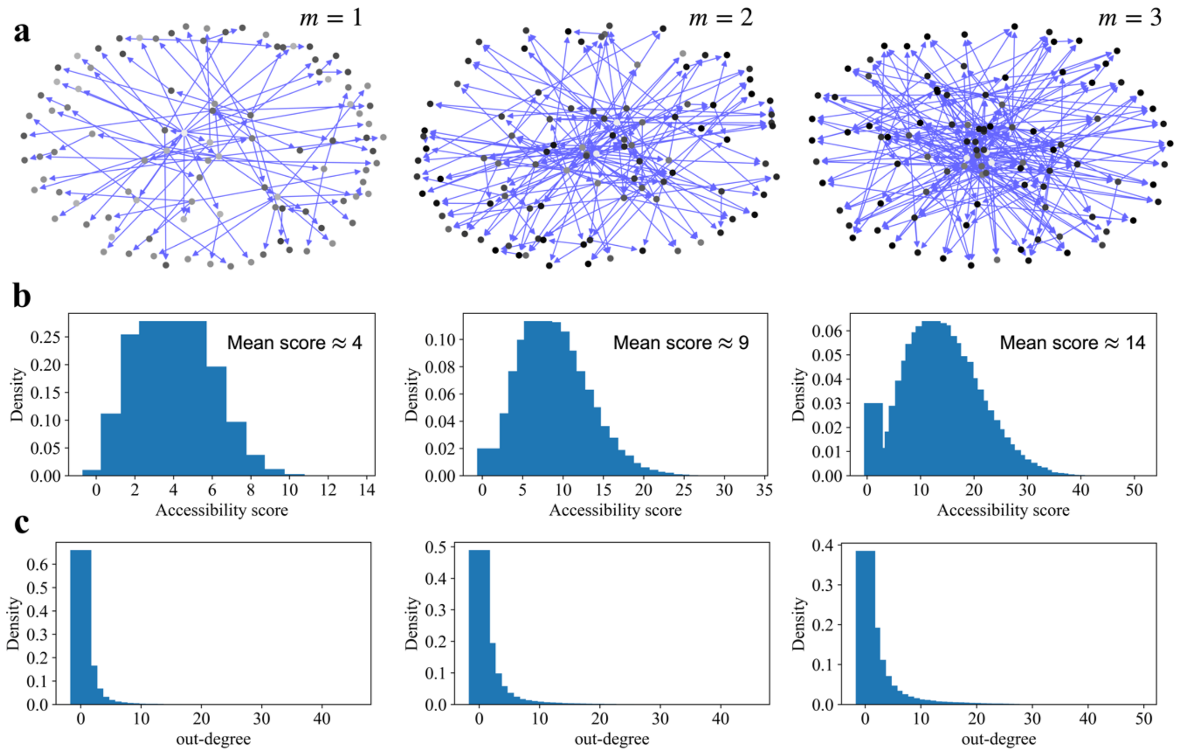


Figure S1: Accessibility scores of nodes in Barabási-Albert graphs as a function of the in-degree to every single node $m$. (**a**) One of 1000 replicates of $m$ =1, 2, and 3 type model GRNs used to survey the accessibility score. (**b**) Distributions of the accessibility scores obtained from 1000 replicates of $m$ =1, 2, and 3 type model GRNs. (**c**) Out-degree distributions for $m$ =1, 2, and 3 type model GRNs from the same 1000 replicates used to compute the accessibility score distributions in Figure S1(b).

Other python packages that were used to compute the loss fields and generate the plots are, numpy, scipy, and matplotlib.

(1) Barabási, A.-L.; Albert, R. Emergence of Scaling in Random Networks. *Science* **1999**, *286* (5439), 509–512. https://doi.org/10.1126/science.286.5439.509.

© 2020 by the authors. Submitted for possible open access publication under the terms and conditions of the Creative Commons Attribution (CC BY) license (http://creativecommons.org/licenses/by/4.0/).
